# Amyloid-β–Induced Changes in Molecular Clock Properties and Cellular Bioenergetics

**DOI:** 10.3389/fnins.2017.00124

**Published:** 2017-03-17

**Authors:** Karen Schmitt, Amandine Grimm, Anne Eckert

**Affiliations:** ^1^Neurobiology Lab for Brain Aging and Mental Health, Transfaculty Research Platform, Molecular and Cognitive Neuroscience, University of BaselBasel, Switzerland; ^2^Psychiatric University Clinics, University of BaselBasel, Switzerland

**Keywords:** Alzheimer's disease, amyloid-β, bioenergetic balance, energetic state, mitochondria

## Abstract

Ageing is an inevitable biological process that results in a progressive structural and functional decline, as well as biochemical alterations that altogether lead to reduced ability to adapt to environmental changes. As clock oscillations and clock-controlled rhythms are not resilient to the aging process, aging of the circadian system may also increase susceptibility to age-related pathologies such as Alzheimer's disease (AD). Besides the amyloid-beta protein (Aβ)-induced metabolic decline and neuronal toxicity in AD, numerous studies have demonstrated that the disruption of sleep and circadian rhythms is one of the common and earliest signs of the disease. In this study, we addressed the questions of whether Aβ contributes to an abnormal molecular circadian clock leading to a bioenergetic imbalance. For this purpose, we used different oscillator cellular models: human skin fibroblasts, human glioma cells, as well as mouse primary cortical and hippocampal neurons. We first evaluated the circadian period length, a molecular clock property, in the presence of different Aβ species. We report here that physiologically relevant Aβ_1–42_ concentrations ranging from 10 to 500 nM induced an increase of the period length in human skin fibroblasts, human A172 glioma cells as well as in mouse primary neurons whereas the reverse control peptide Aβ_42-1_, which is devoid of toxic action, did not influence the circadian period length within the same concentration range. To better understand the underlying mechanisms that are involved in the Aβ-related alterations of the circadian clock, we examined the cellular metabolic state in the human primary skin fibroblast model. Notably, under normal conditions, ATP levels displayed circadian oscillations, which correspond to the respective circadian pattern of mitochondrial respiration. In contrast, Aβ_1–42_ treatment provoked a strong dampening in the metabolic oscillations of ATP levels as well as mitochondrial respiration and in addition, induced an increased oxidized state. Overall, we gain here new insights into the deleterious cycle involved in Aβ-induced decay of the circadian rhythms leading to metabolic deficits, which may contribute to the failure in mitochondrial energy metabolism associated with the pathogenesis of AD.

## Introduction

Ageing leads to a functional deterioration of many brain systems, including the circadian clock, an internal time-keeping system that generates ~24-h rhythms in physiology and behavior. Healthy as well as pathological brain aging is associated with disturbances in both sleep-wake cycle and circadian rhythms (Froy, 2011; Kondratova and Kondratov, 2012; Musiek and Holtzman, 2016). Age-related circadian abnormalities have generally been considered as consequences of neurodegenerative processes. However, it has been recently implied that circadian disruptions can exacerbate the severity of several age-related neurodegenerative pathologies. AD is the most common neurodegenerative disorder among elderly individuals. It accounts for up to 80% of all dementia cases and ranks as the fourth leading cause of death among those above 65 years of age. Multiple clinical studies have demonstrated that the disruption of sleep and circadian rhythms is one of the common and earliest signs of AD. Thus, abnormalities in the circadian clock and in sleep quality worsen as the disease progresses (Hofman and Swaab, 2006; Wulff et al., 2010). Although this link between AD and circadian system is increasingly evident, the underlying mechanisms are still not well understood. Potential molecular mechanisms include the circadian control of physiological processes such as brain metabolism, amyloid-β (Aβ) metabolism, reactive oxygen species (ROS) homeostasis, hormone secretion, autophagy, and stem cell proliferation (Hastings et al., 2007; Kang et al., 2009; Lai et al., 2012; Eckel-Mahan and Sassone-Corsi, 2013). However, it remains mostly unclear if or how Aβ might lead to disruption of the circadian clock which, in turn, could exacerbate the neurodegenerative processes. As the underlying mechanisms of AD onset are still unknown, it is worth considering the core clock as a potential therapeutic target for the prevention of neurodegeneration.

Although the exact molecular nature of AD pathogenesis is still unclear and widely debated, a growing body of evidence supports mitochondrial dysfunction as a prominent and early, chronic oxidative stress-associated event that contributes to synaptic abnormalities and ultimately, selective neuronal degeneration in AD (Schmitt et al., 2012). The mitochondrial energy deficiency is a fundamental characteristic of the AD brain (Manczak et al., 2004) as well as of peripheral cells derived from AD patients (Gibson et al., 1998). Since cellular function relies heavily on this organelle, alterations to the mitochondrial function have severe consequences on neuronal activity and survival, which can, at least in part, contribute to the development of AD.

It is essential to better understand the underlying molecular mechanisms between the circadian network and the AD pathology, particularly in regards to Aβ-related mitochondrial alterations. In this study, we show evidence that Aβ (i) likely contributes to the circadian clock disruption in AD at the molecular level and (ii) impairs the circadian oscillations of the mitochondrial activity with regard to oxygen consumption and ATP generation. Our findings are consistent with the existence of a crosstalk between the clock and the mitochondrial network that maintains bioenergetic homeostasis in response to circadian metabolic changes as well as with the Aβ-related mitochondrial cascade hypothesis.

## Materials and methods

### Chemicals and reagents

Dulbecco's-modified Eagle medium (DMEM), RPMI-1640 medium, fetal calf serum (FCS), penicillin/streptomycin, NAD^+^, NADH, dihydrorhodamine 123 (DHR), HBSS, MTT [3-(4,5-Dimethylthiazol-2-yl)-2,5-diphenyltetrazolium bromide] and PES (phenazine ethosulfate) were from Sigma-Aldrich (St. Louis, MO, USA). Glutamax and DPBS were from Life Technologies (Waltham, MA, USA). XF Cell Mitostress kit was from Seahorse Bioscience (North Billerica, MA, USA). Horse serum (HS) was from Amimed, Bioconcept (Allschwil, Switzerland).

### Cell culture and synchronization of the circadian rhythms

Isolated human skin fibroblasts from biopsies and glioma cell line A172 (gift from Dr Steven A. Brown) were cultured in DMEM/1% penicillin-streptomycin (v/v)/1% Glutamax (v/v) [DMEM complete (DMEMc)]/20% heat-inactivated FBS (v/v) (Pagani et al., 2011; Gaspar and Brown, 2015). Confluent cells were infected using mice Bmal1::luciferase lentivirus and were positively selected 3 days after infection. Mouse cortical and hippocampal neurons were prepared from E15 embryos according to the Swiss guidelines and were plated in poly-l-lysine-coated plates according to the instruction of the manufacturer (Lonza, Switzerland). After 7 days at 37°C, 50% of the medium was replaced with fresh medium every third day.

Prior to the assessment of circadian period length, cells were synchronized for 15 min at 37°C with 100 mM dexamethasone as described previously (Pagani et al., 2011), while prior to the measurement of nucleotide levels, mitochondrial reactive oxygen species (mROS) and oxygen consumption rate (OCR) in human primary fibroblast culture, serum shock treatment [DMEMc supplemented with 50% heat-inactivated horse serum (v/v)] was performed for 2 h at 37°C to synchronize the cells accordingly to a previously optimized method for these kind of experiments (Cooper, 2003; Rosner et al., 2013). For measurements of nucleotides levels and mitochondrial reactive oxygen species (mROS) levels, experiments were performed starting from 12 h post-synchronization time point and measured at 4 h intervals. For measurements of oxygen consumption, experiments were performed at 16 and 28 h post-shock.

### Amyloid-beta peptide preparation and treatment

The different amyloid-beta (Aβ) fragments [Aβ_1–42_; reverse peptide (Aβ_42−1_); Aβ_1–40_; N-terminal fragment (Aβ_1–28_); C-terminal fragment (Aβ_34–42_); shortest active peptide fragments (Aβ_25–35_, Aβ_15–25_)] (Bachem, Bubendorf, Switzerland) were rapidly dissolved in sterile PBS 1x, pH~7.4 (stock concentration 500 μM) and stored as aliquots at −80°C until needed. One day before treatment of cells with Aβ peptide fragments, 50 μM Aβ working solutions were prepared in PBS 1x, pH~7.4. After securing the caps with parafilm, the tubes were incubated on a table-top thermomixer (Eppendorf, Hamburg, Germany) at 1,000 rpm (~250 × g) at 37°C for 24 h in order to induce aggregation of the Aβ peptides into fibrils. For the circadian period length determination, after synchronization with dexamethasone (100 nM, 15 min at 37°C), fibroblasts were treated with 1, 10, 250, and 500 nM of Aβ in DMEM cell culture medium containing 1% penicillin-streptomycin (v/v)/1% Glutamax (v/v) (DMEMc)/10% FBS (v/v) for 5 days until analyzed. This experimental setting requesting a rather long incubation time over several days in aqueous medium, predicting the formation of insoluble fibrillar Aβ aggegrates and fibrils, did not allow to study the effects only of soluble Aβ species. Therefore, also in the following assays, the same Aβ preparations were used for the determination of nucleotides, ROS and oxygen consumption rate to mimic corresponding conditions of Aβ assembly and chronic toxicity. Thus, the cells were pre-treated with 500 nM pre-aggregated Aβ (the most effective concentration on bioenergetics readouts without cell death-inducing properties) in DMEM cell culture medium containing 1% penicillin-streptomycin (v/v)/1% Glutamax (v/v) [DMEM complete (DMEMc)]/20% heat-inactivated FBS (v/v) for 5 days. After the synchronization, cells were treated again with 500 nM Aβ in DMEM/1% penicillin-streptomycin (v/v)/1% Glutamax (v/v) (DMEMc)/2% FBS (v/v) up to 48 h to maintain the Aβ pressure on the synchronized cells.

### Circadian period length measurement

Human skin fibroblasts transfected with the lentiviral circadian reporter mice Bmal1::luciferase (Brown et al., 2005; Gaspar and Brown, 2015) were plated in single culture dishes (35 × 10 mm) at a cell density sufficient to reach 70–80% of confluency on the start day of recording of period length. After synchronization with dexamethasone (100 nM, 15 min at 37°C), cells were cultured in DMEM supplemented with 10% heat-inactivated FBS, 10 mM HEPES and 0.1 mM luciferin. For at least 5 days, the amount of produced light that is proportional to Bmal1 gene expression was measured using a Lumicycle instrument (Actimetrics). Data were analyzed with Lumicycle Analysis software (Lumicycle™, version 2.31, Actimetrics Software) and the period of oscillation was calculated by least-mean-squares fitting of dampened sine wave functions to the actual data as described previously (Pagani et al., 2011).

### Nucleotides measurements

The total ATP content from synchronized human skin fibroblasts was determined using a bioluminescence assay (ViaLighTM HT; Cambrex Bio Science, Walkersville, MD USA) according to the instruction of the manufacturer. Cells were plated in 8 replicates into a 96-wells cell culture plate at a cell density sufficient to reach 40–50% of confluency on the following day. The enzyme luciferase, which catalyzes the formation of light from ATP and luciferin was used. The emitted light is linearly related to the ATP concentration and is measured using a multilabel plate reader VictorX5 (Perkin Elmer).

To measure NAD^+^ and NADH, the two molecules were separately extracted using an acid-base extraction method (HCL 0.1 mol/l–NAOH 0.1 mol/l). To determinate both NAD^+^ and NADH, an assay was used that is based on passing the electron from ethanol through reduced pyridine nucleotides to MTT [3-(4,5-Dimethylthiazol-2-yl)-2,5-diphenyltetrazolium bromide] in a PES (phenazine ethosulfate)-coupled reaction resulting in the formation of a purple precipitate (formazan) that, once dissolved, can be quantified at 595 nm (VictorX5, Perkin Elmer).

### Oxygen consumption rate (OCR)

The OCR was evaluated in synchronized fibroblasts as previously described (Invernizzi et al., 2012). Briefly, human skin fibroblasts were seeded at the density of 3 × 10^4^ cells/100 μl per well on Seahorse Biosciences 24-well culture plates 1 day prior to the serum-shock synchronization. On the next day, cells were synchronized using serum shock (2 h at 37°C). Afterwards, the medium was exchanged to 500 μl of assay medium [glucose-free RPMI-1640 medium containing 2% FBS (v/v), 2 mM sodium pyruvate, pH~ 7.4]. Before to be placed in the Seahorse XF24 Analyzer, the microplates were equilibrated in a CO_2_-free incubator at 37°C for 60 min. Then, the OCR allocated to basal respiration at 16 and 28 h after synchronization were recorded in real-time over 30 min.

### Mitochondrial reactive oxygen species (mROS) determination

Total level of mROS was assessed using the fluorescent dye dihydrorhodamine 123 (DHR). Cells were plated in 8 replicates into a black 96-wells cell culture plate at a cell density sufficient to reach 40–50% of confluency on the day of the start of mROS recording. After synchronization, cells were loaded at the indicated time points with 10 μM of DHR for 15 min at room temperature in the dark on an orbital shaker. After washing twice with HBSS, DHR, which is oxidized to cationic rhodamine 123 that localizes within the mitochondria, exhibits a green fluorescence that was detected using the multilabel plate reader VictorX5 at 485 nm (excitation)/538 nm (emission). The intensity of fluorescence was proportional to mROS levels in mitochondria.

### Statistical analysis

Data were presented as mean ± S.D. Statistical analyses were performed using the GraphPad Prism software. The curves were generated by using a standard curve fit function in the GraphPad Prism software. For statistical comparisons, One-way ANOVA followed by Tukey's multiple comparison test or Two-way ANOVA followed by Bonferroni's multiple comparison test was used respectively. *P* < 0.05 were considered statistically significant. Rhythmicity of ATP, NAD^+^, NADH, and mROS was assessed using a non-parametric algorithm previously described for determination of rhythmic transcripts (Hughes et al., 2010). The JTK-cycle algorithm was used as implemented in R by Kronauer as previously described (Dallmann et al., 2012). A window of 24 h was used for the determination of circadian periodicity and a *P*-value of < 0.05 was considered as statistically significant.

## Results

### Aβ disturbs circadian period length *in vitro*

**Figure 1:**
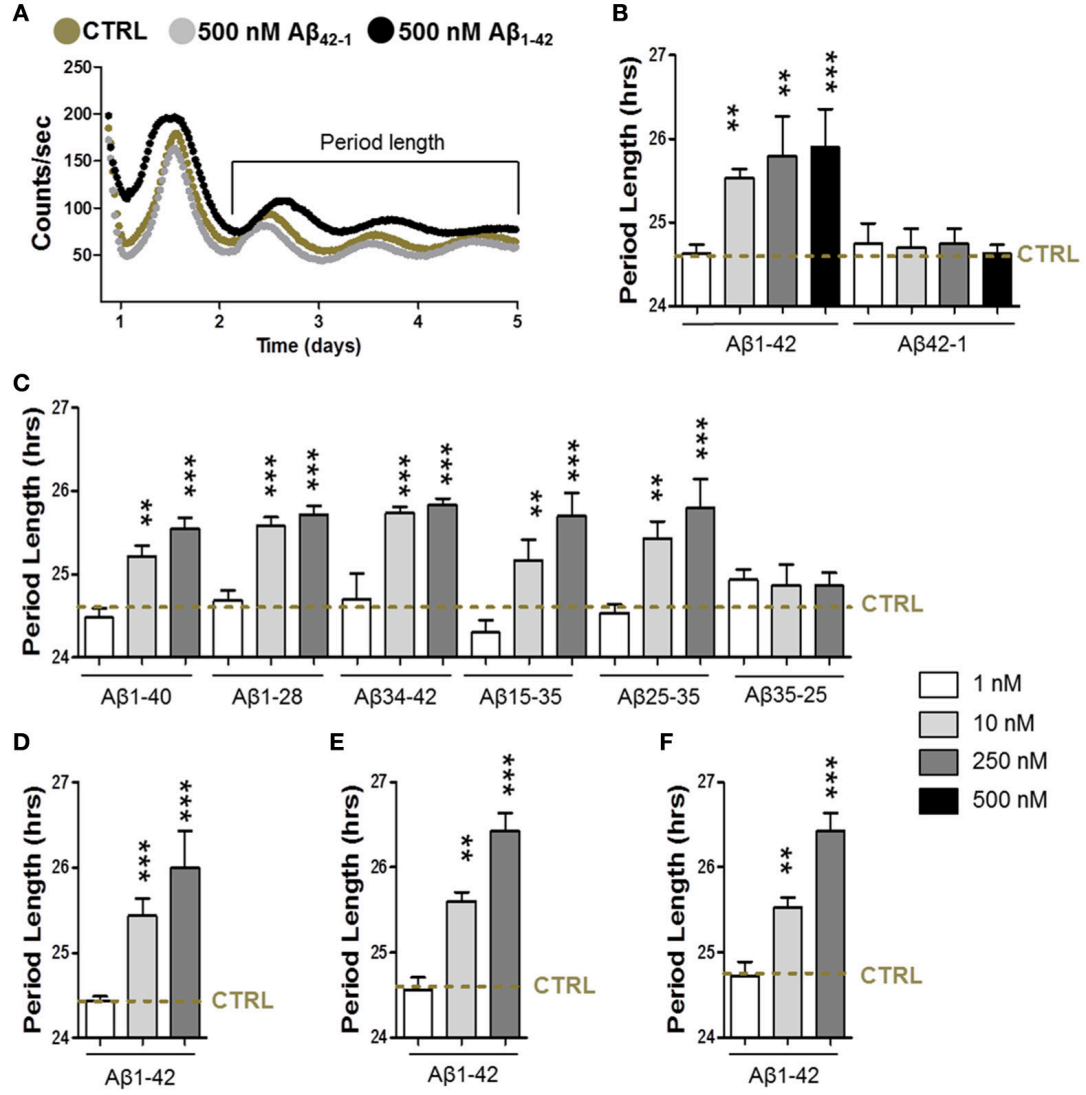
**Amyloid-β increases the circadian period length. (A)** Representative luminescence records from human skin fibroblasts transfected with mice Bmal1::luciferase reporter in presence of amyloid-β 1–42 (500 nM) or of the reverse peptide amyloid-β 42-1 (500 nM) compared to the condition in the absence of Aβ species (CRTL). **(B, C)** Circadian period length determined in synchronized human skin fibroblasts transfected with mice Bmal1::luciferase reporter in presence of amyloid-β 1–42 and 42-1 **(B)**, 1–40, 1–24, 34–42, 25–35, 35-25, and 15–25 **(C)** compared to the condition in the absence of Aβ species (CRTL) (dashed line). **(D)** Circadian period length determined in synchronized human glioma A172 cells transfected with Bmal1::luciferase reporter in presence of amyloid-β 1–42 compared to control (CRTL) (dashed line). **(E)** Circadian period length determined in synchronized cortical primary mouse neurons transfected with Bmal1::luciferase reporter in presence of amyloid-β 1–42 compared to control (CRTL) (dashed line). **(F)** Circadian period length determined in synchronized hippocampal primary mouse neurons transfected with Bmal1::luciferase reporter in presence of amyloid-β 1–42 compared to control (CRTL) (dashed line). Bars represent the mean of at least three independent experiments ± SD. ^**^P < 0.01; ^***^P < 0.001 for Student's two-tailed *t*-test, compared to control.

To address the question whether Aβ is able to disturb the circadian function, we first characterized the circadian period length of synchronized fibroblasts in the presence of different Aβ fragments (Figures 1A–C). For that purpose, we infected cells with a circadian reporter construct (Bmal1 promoter-driven expression of firefly luciferase-harboring lentivirus). First, the effect of Aβ_1–42_, the major component of amyloid plaques in AD brains that is more aggregation prone and exhibits higher neurotoxicity than Aβ_1–40_, was investigated. Notably, cells had a significantly longer period length in the presence of aggregated Aβ_1–42_ at concentrations from 10 up to 500 nM compared to controls (Figures 1A,B), while 1 nM was not yet effective. At a concentration of 250 nM the maximum effect on prolongation of period length was already reached. However, amplitude (the difference between peak and nadir expression values) was unchanged (data not shown). Notably, up to a concentration of 500 nM, cell viability was not significantly impaired in these experimental settings (data not shown). Consistent with findings showing that the reverse control peptide Aβ_42*-*1_ is inactive (Zhang et al., 2002), Aβ_42*-*1_ did not disturb the cellular circadian properties. Similarly, Aβ_1–42_ treatment also induced an increase in the period length in synchronized glioma A172 cells as well as in cortical and hippocampal primary mouse neurons (Figures 1D–F). In order to identify the specific Aβ fragment/s responsible for the increase in the period length, we then treated the synchronized fibroblasts with different Aβ species (Aβ_1–40_, the most abundant Aβ isoform in the brain, Aβ_1–28_, Aβ_34–42_, Aβ_15–25_, and Aβ_25–35_) within the same range of concentrations (Figure 1C). Surprisingly, all the fragments induced a significant alteration of the circadian function to a similar extent as Aβ_1–42_ did itself. Together, these results suggest that the aggregated nature of the Aβ species seem to represent an important factor for the AD-related disruption of the circadian rhythm.

### Aβ induces a decline in the circadian bioenergetic homeostasis

**Figure 2:**
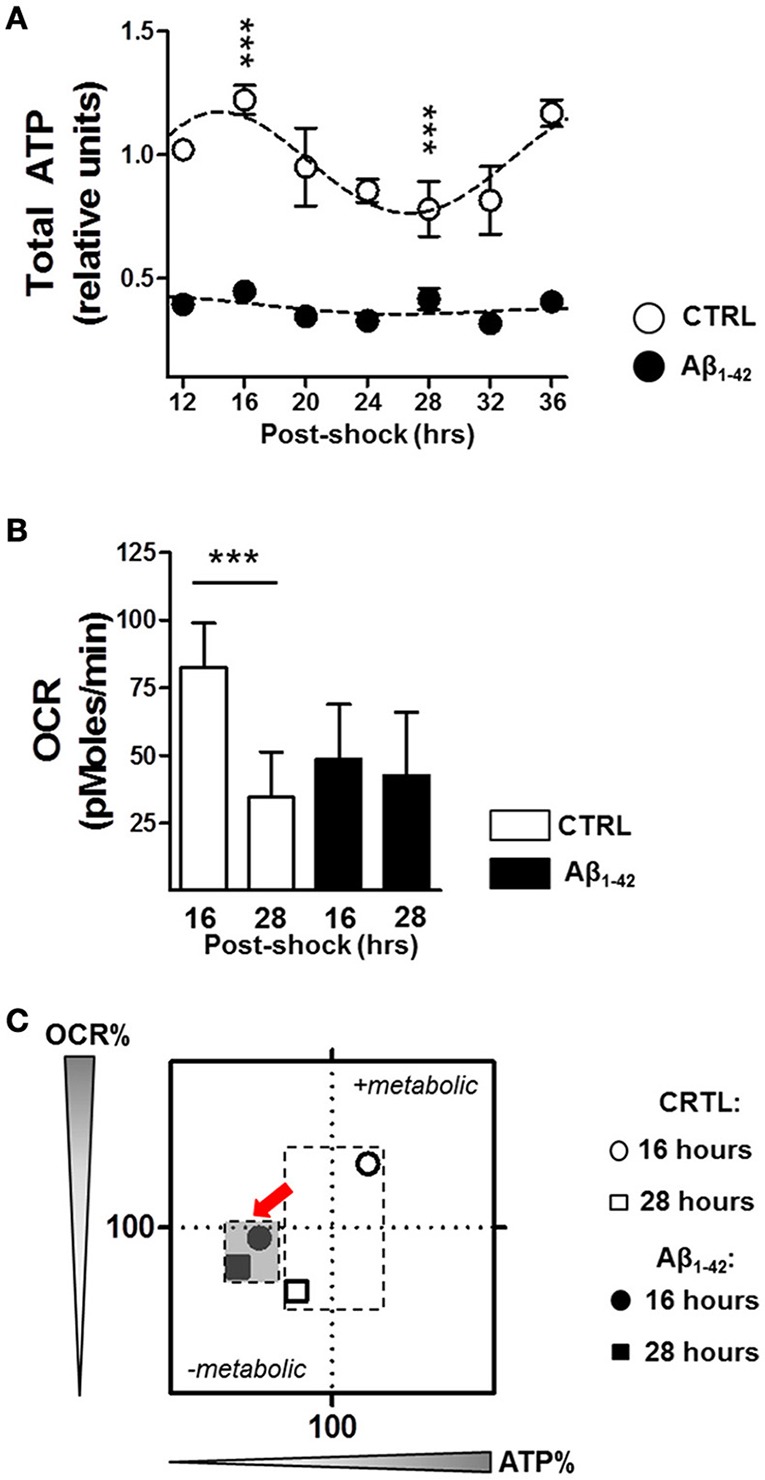
**Aβ induces a decline in circadian bioenergetic homeostasis. (A)** Total ATP levels from synchronized human skin fibroblasts treated with Aβ 1–42 compared to non-treated cells (CTRL) measured at the indicated time points (6 time points, *n* = 6 for each) (Two-way ANOVA; Aβ effect: *Df* = 1, *F* = 3890, *P* < 0.0001; time effect: *Df* = 6, *F* = 34.25, *P* < 0.0001; *post hoc* Bonferroni, ctrl vs. Aβ: at 16 h, *t* = 23.43, ^***^P < 0.0001, at 28 h, *t* = 11, ^***^P < 0.0001). Rhythmicity of ATP was assessed using the JTK_Cycle algorithm as previously described (Dallmann et al., 2012): JTK_Cycle, P_CTRL_ = 2.07 × 10^−15^, P_*Aβ*_ = 0.691. **(B)** Oxygen Consumption Rate (OCR) was evaluated in Aβ-treated human skin fibroblasts compared to untreated control cells at 16 and 28 h after synchronization, respectively. (*n* = 11 replicates /group, three independent experiments) (Two-way ANOVA, Aβ effect: *Df* = 1, *F* = 9.948, *P* = 0.0021; time effect: *Df* = 1, *F* = 44.08, *P* < 0.0001; *post hoc* Bonferroni, ctrl vs. Aβ: at 16 h, *t* = 5.886, ^***^P < 0.0001, at 28 h, *t* = 1.448, *P* > 0.05). **(C)** OCR/ATP: The basal OCR corresponding to the ATP peak (at 16 h) and the ATP trough (at 28 h) are plotted against the respective ATP content in control (white symbols) and Aβ conditions (black symbols). Values represent the mean of each group (mean of the ATP levels in abscissa/mean of the OCR in ordinate); *n* = 11–12 replicates/group of three independent experiments. The red arrows highlight the Aβ-induced metabolic shift at 16 and 28 h after cell synchronization.

Since mitochondria were found to be a target of Aβ (Schmitt et al., 2012), leading to mitochondrial dysfunction including a decline in OxPhos and ultimately in ATP content, we investigated the impact of Aβ on ATP content and on mitochondrial oxidative metabolism with regard to rhythmic changes due to the the tight relationship between the circadian clock and metabolism (Brown, 2016; Panda, 2016). As ATP is primarily generated via mitochondrial oxidative phosphorylation (OxPhos), we first monitored whole cell ATP content as readout of OxPhos in Aβ_1–42_-treated (500 nM) compared with untreated synchronized human skin fibroblasts. Under normal conditions, total ATP levels displayed a circadian rhythmicity in control cells (with the peak at 16 h and the trough at 28 h), whereas ATP levels in the Aβ-treated cells did not oscillate and were, in addition, significantly reduced (Figure 2A).

We then monitored the real–time oxygen consumption rate (OCR), an indicator of mitochondrial respiration in Aβ-treated human skin fibroblasts compared to untreated cells at 16 h corresponding to the ATP peak and at 28 h related to the ATP trough using the Seahorse Bioscience XF24 Flux Analyzer (Figure 2B). In untreated control cells, the OCR was significantly decreased at the time point of the ATP trough (at 28 h) compared to that of the ATP peak (at 16 h) (Figure 2B). Thus, the circadian oscillations of OCR and ATP appear to be in the same phase and fit well together in the way that at the time point of high energy production, the oxidative phosphorylation system provides the maximum performance. In contrast, Aβ_1–42_ treatment completely dampened the variation in the OCR and no differences were found at 16 and 28 h after synchronization (Figure 2B). Overall, these observations support the hypothesis that circadian control of the mitochondrial function is strongly disturbed by Aβ which may lead to a mitochondrial inability to respond to an increasing metabolic demand. In addition, we characterized the cellular bioenergetic profile of Aβ-treated human skin fibroblasts compared to untreated cells, by mapping OCR (basal respiration) vs. ATP for the indicated time points (Figure 2C). Remarkably, untreated cells switched between a metabolically active state corresponding to the high ATP (16 h) and a metabolically resting state corresponding to low ATP levels (28 h), while Aβ-treated cells remained in the metabolically inactive state at both time points.

### Alterations in Aβ-related mitochondrial functions lead to oxidative stress

**Figure 3:**
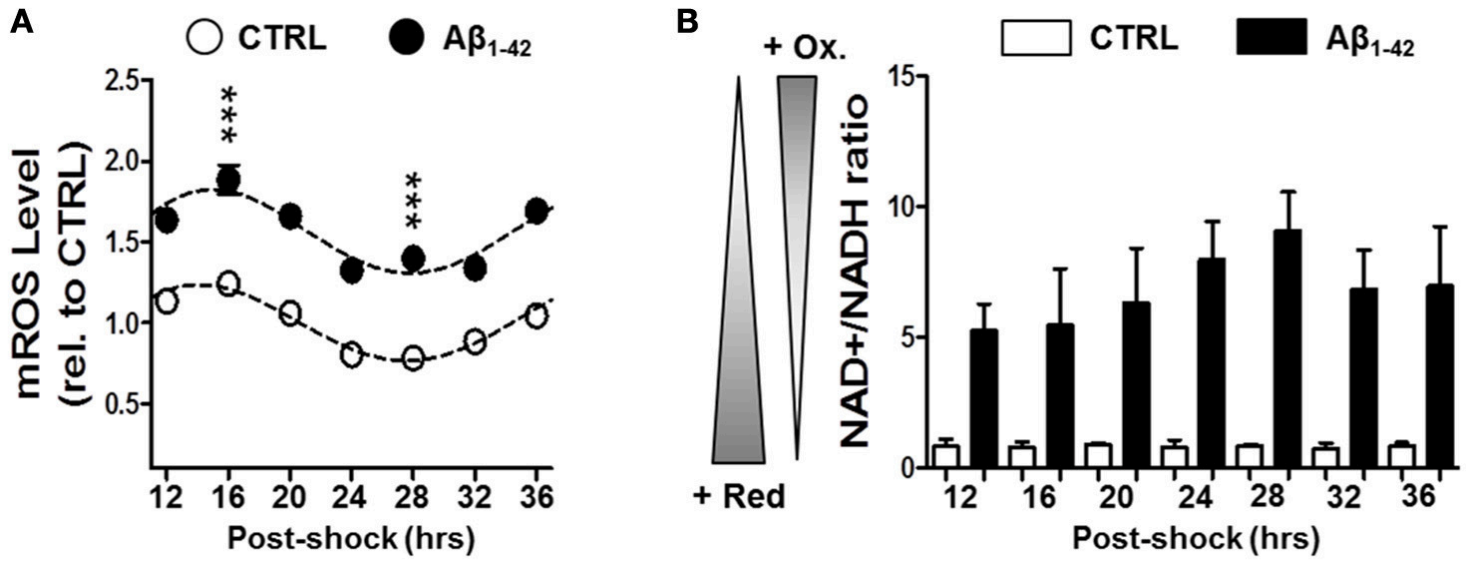
**Aβ induces a disruption in the cellular redox state. (A)** Mitochondrial (mROS) reactive oxygen species levels were evaluated in synchronized human skin fibroblasts treated with Aβ 1–42 compared to non-treated cells (CTRL) measured at the indicated time points (6 time points, *n* = 4 for each) (Two-way ANOVA; Aβ effect: *Df* = 1, *F* = 859, *P* < 0.0001; time effect: *Df* = 6, *F* = 33.16, *P* < 0.0001; *post hoc* Bonferroni, ctrl vs. Aβ: at 16 h, *t* = 9.757, ^***^P < 0.0001, at 28 h, *t* = 9.035, ^***^P < 0.0001). Rhythmicity of mROS was assessed using the JTK_Cycle algorithm as previously described (Dallmann et al., 2012): JTK_Cycle, P(CTRL)_mROS_ = 5.40 × 10^−21^, P(Aβ)_mROS_ = 3.9 × 10^−18^. **(B)** The NAD^+^/NADH ratio evaluated in in synchronized Aβ-treated human skin fibroblasts compared to untreated control cells at the indicated time points. Values represent the mean ± SD; *n* = 9–12 replicates of three independents experiments (Two-way ANOVA; Aβ effect: *Df* = 1, *F* = 408.7, *P* < 0.0001; time effect: *Df* = 6, *F* = 1.769, *P* = 0.1343).

To further investigate the Aβ-induced imbalance in mitochondrial homeostasis, we next evaluated the mitochondrial ROS levels as well as the NAD^+^/NADH ratio as oxidative stress readout in synchronized human skin fibroblasts in the presence or absence of Aβ (Figure 3). Again under normal conditions, we found circadian oscillations for mitochondrial ROS (mROS) levels exhibiting a peak at 16 h and a trough at 28 h after synchronization consistent with our findings on ATP oscillations. Interestingly, Aβ_1–42_ (500 nM) increased globally the mROS levels in the Aβ-treated cells, but it did not induce a dampening of the circadian rhythmicity compared to the untreated cells as found for bioenergetic readouts (Figure 3A). In contrast, the NAD^+^/NADH ratio did not exhibit a circadian pattern in control cells (Figure 3B). However, the ratio was significantly augmented by Aβ_1–42_ treatment (about 10 times) compared to untreated cells (Figure 3B). Together, our findings suggest that Aβ triggers a decline of mitochondrial bioenergetics including an abolishment of variations in ATP levels coupled to a shift in the redox homeostasis toward a highly oxidized state.

## Discussion

In our study, we aimed to investigate, on the one hand, whether Aβ can directly play a role in the molecular circadian clock disturbances associated with AD and on the other hand, whether Aβ impacts the integrity of the circadian regulation of mitochondrial function, which could, in part, contribute to the AD pathogenesis. The major findings were that, (i) AD-related Aβ species are able to induce alterations in the molecular circadian rhythms; (ii) Aβ provoked a drastic dysregulation in the circadian control of the mitochondrial respiratory capacity including reduced energy levels along with increased ROS production.

Given that circadian rhythms become weaker and less synchronized in specific regions of the brain (i.e., SCN) with aging (Farajnia et al., 2012; Hastings and Goedert, 2013), the progressive desynchrony likely promotes Aβ aggregation contributing to the pathogenesis of AD. Comparable to human studies (Weldemichael and Grossberg, 2010), it was shown for several animal models of AD that they exhibit disturbances of behavioral and physiological circadian rhythms (Musiek, 2015). In mice, the circadian disturbances appear to correlate with the degree of amyloid plaque burden and it has been suggested that aggregated forms of Aβ might disrupt the circadian clock (Sterniczuk et al., 2010; Roh et al., 2012). One proposed explanation of circadian dysfunction in AD is the Aβ-related impairment of the SCN caused by the drastic loss of vasopressin- and vasoactive intestinal peptide-expressing neurons which are essential in the maintenance of the SCN circadian function (Swaab et al., 1985; Zhou et al., 1995; Farajnia et al., 2012).

Moreover, considerable evidence has emerged linking the sleep-wake cycle with Aβ regulation in the brains of mice and humans (Kang et al., 2009; Huang et al., 2012; Lim et al., 2014). The diurnal variation of Aβ levels suggests that neuronal activities related to the sleep/wake cycle directly influence levels of Aβ in the brain (Nir et al., 2011). Synaptic activity has been shown to increase interstitial fluid Aβ release from neurons in both mice and men (Cirrito et al., 2005; Brody et al., 2008). Similarly, sleep deprivation increases plaque formation (Kang et al., 2009), whereas enhanced sleep reduces Aβ deposition (Tabuchi et al., 2015). The most active brain regions during quiet wakefulness are located in the default mode network (DMN) and have been reported as the first network affected by AD (Greicius et al., 2004) exhibiting the most Aβ deposition during the development of AD pathology (Mormino et al., 2011). Consistent with observations of an alteration of circadian behavior in MCI and AD patients, it can be speculated that the effects of Aβ protein on the circadian period length can be related to disturbances in the circadian rhythm and the sleep/wake cycle in MCI and early-stage AD patients where a functional disruption of the SCN is even observed in early disease stages. These age-associated changes in the SCN have been proposed to be responsible for the impairment of circadian clock synchronization throughout the body causing circadian and sleep defects (Zhou et al., 1995; Stopa et al., 1999).

Indeed, physiologically relevant concentrations of Aβ_1–42_, which were not yet cytotoxic, were able to alter circadian rhythms by increasing the period length in human fibroblasts, human A172 glioma cells as well as in mouse primary neurons. Unexpectedly, similar effects were detected with different synthetic Aβ peptides including peptides lacking the N- and C-terminus of the Aβ sequence (Aβ_1–28_, Aβ_34–42_, Aβ_25–35_, and Aβ_15–25_). The whole amino-acids sequence of Aβ_1–42_ seems to be involved in the disruption of the circadian rhythm (Mariani et al., 2007), but already short Aβ fragments such as Aβ_25–35_ which is processed *in vivo* by brain proteases (Clementi et al., 2005), retain the effect of the full-length peptide on the period length in our *in vitro* experiments, while Aβ fragments exhibiting the reverse amino acid sequence were devoid of any action (Aβ_42*-*1_ and Aβ_35–25_). Unlike the reverse Aβ peptides, several peptide fragments of Aβ, including our selection of Aβ species, form aggregates *in vitro* which have a similar fibrillary morphology in aqueous buffers (Serpell, 2000). Hence, the structure and the assembly of the Aβ protein might be crucial for its mode of action on circadian rhythmicity such as that of the period length.

Consistent with a growing body of evidence on the circadian regulation of the mitochondrial function (Manella and Asher, 2016), metabolic byproducts of mitochondrial metabolism including ATP and mROS exhibited circadian rhythmicity. Concurrent to the circadian rhythmicity in ATP levels, mitochondrial oxygen consumption analyses showed time-of-day-dependent variation in basal respiration (Isobe et al., 2011; Peek et al., 2013). When in resting state (low ATP), observed 28 h post-synchronization in cultured cells, the cells exhibited a low respiration as well. The reverse was seen at 16 h post-synchronization, when the cells showed high ATP levels and high respiration respectively. Thus, the circadian patterns of OCR, ATP and mROS seem likely to be in the same phase and fit well together in the way that at the time point of high energy production the oxidative phosphorylation system provides the maximum performance accordingly to the high energy demand, initiating at the same time a maximum in mROS production due to the high electron leakage from the electron transport chain. Surprisingly, we did not observe a circadian rhythmicity of the NAD^+^/NADH ratio, although it has been showed that this ratio exhibited distinct circadian rhythms in ApoE^−/−^ and C57BL/6J SCN under the constant dark condition (Zhou et al., 2016). The difference with our *in vitro* observations might be possibly due to the fact that the SCN is a stronger clock than fibroblasts as peripheral oscillators (Welsh et al., 2004).

Since disturbances in mitochondrial bioenergetics are known as early and prominent features of AD-related neuronal toxicity induced by Aβ (Yao et al., 2009; Garcia-Escudero et al., 2013), we examined the potential impacts of Aβ on the integrity of the mitochondrial function. Concomitantly, defects in the mitochondrial function induced a decline in mitochondrial metabolism including decreased mitochondrial respiration, depletion in ATP content and increased oxidative stress through increased mitochondrial ROS levels and drastic changes in the NAD^+^/NADH ratio. Interestingly, only the rhythmicity of ATP and OCR was entirely dampened in the presence of Aβ. This Aβ-related loss of rhythmicity in basal respiration and energy production could be explained by altered expression patterns of the circadian clock genes caused by an Aβ-induced post-translational degradation of the circadian clock genes, Bmal1, and Per2 (Song et al., 2015), thereby probably impairing the expression of clock-controlled genes involved in the oxidative phosphorylation (Panda et al., 2002). This impairment may, in turn lead to alterations of the rhythmic assembly of mitochondrial supercomplexes to reach their maximum performance in OXPHOS. Moreover, in addition to the Aβ-related alterations in numerous enzymes essential for the mitochondrial functions (Gibson et al., 1998), it has been also recently proposed that the PERIOD proteins serve as a rheostat for mitochondrial nutrient utilization by regulating rate-limiting mitochondrial enzymes and therefore improving mitochondrial metabolism (Neufeld-Cohen et al., 2016). Overall, these observations suggest that Aβ likely induces alterations in the molecular clock gene expression, which lead, in turn, to changes in clock-controlled gene expressions and eventually to disturbances in mitochondrial respiration and in energy production.

Given the role of the circadian system in the regulation of ROS homeostasis (Kondratova and Kondratov, 2012; Lee et al., 2013), it is surprising that, while mROS levels increased drastically in the presence of Aβ, they retained their circadian rhythmicity despite the proposed Aβ-related alterations in the molecular clock. Indeed, it has been described that ROS levels oscillated in different mouse tissues and these rhythmic patterns were disrupted in mice bearing impaired circadian clocks (Kondratov et al., 2006). Our findings could be explained, at least in part, by the fact that fibroblasts are known to have stronger antioxidant defense mechanism than the brain (Cecchi et al., 2002). Other explanations might be that the expression of clock-controlled genes regulating the cellular antioxidant defense are less sensitive to Aβ-induced insults than those of the OXPHOS or that the antioxidant defense enzymes may act upstream of the electron transport chain which is disturbed in its function by Aβ in this way initiating and increasing oxidative stress. Further research is needed to elucidate the underlying molecular mechanisms of these cellular response systems. Our findings are in agreement with previous observations from both animal models and AD patients exhibiting mitochondrial failure as well as elevated oxidative stress in their brains (Cecchi et al., 2002; Shi and Gibson, 2007; Zhu et al., 2013).

In summary, we gained new insights into the Aβ-related molecular circadian impairments in AD. Based on the complexity of the multifactorial nature of AD, several vicious cycles are interconnected within a larger vicious cycle where mitochondria play a prominent role in the cascade of events leading to AD. All of them, once set in motion, amplify their own processes, thus accelerating the development of AD. One of these subordinate cycles may represent the impact of a disrupted circadian rhythm on mitochondrial function. Finally, the critical role of mitochondria in the early pathogenesis of AD may make them attractive as a preferential target for therapeutic strategies by sustaining mitochondrial metabolic function. Since the diurnal oscillations of Aβ levels in the brain appear to be closely related to the sleep-wake cycle and Aβ, in turn, impacts the circadian regulation of mitochondrial functions, the possibility exists that treating the aging-related sleep-wake impairments and upstream events of the circadian dysfunction early, even prior to the development of AD pathology, might prevent or slow down AD pathogenesis pathways.

## Author contributions

KS and AG performed experiments. AE conceived the project, coordinated and supervised research. KS and AE wrote the manuscript.

## Funding

This work was supported by Swiss National Science Foundation (#31000_122572 and #31003A_149728), Novartis Foundation for Biomedical Research Basel, Synapsis Foundation and the Fonds der Freiwilligen Akademischen Gesellschaft Basel (all to AE).

### Conflict of interest statement

The authors declare that the research was conducted in the absence of any commercial or financial relationships that could be construed as a potential conflict of interest.
